# ICTV Virus Taxonomy Profile: Hepeviridae 2022

**DOI:** 10.1099/jgv.0.001778

**Published:** 2022-09-28

**Authors:** Michael A. Purdy, Jan Felix Drexler, Xiang-Jin Meng, Heléne Norder, Hiroaki Okamoto, Wim H. M. Van der Poel, Gábor Reuter, William M. de Souza, Rainer G. Ulrich, Donald B. Smith

**Affiliations:** 1 Centers for Disease Control and Prevention, Atlanta, GA, USA; 2 Charité-Universitätsmedizin Berlin, Corporate Member of Freie Universität Berlin, Humboldt-Universität zu Berlin, Institute of Virology, Berlin, Germany; 3 Virginia Polytechnic Institute and State University, Blacksburg, VA, USA; 4 University of Gothenburg, Gothenburg, Sweden; 5 Sahlgrenska University Hospital, Gothenburg, Sweden; 6 Jichi Medical University School of Medicine, Tochigi, Japan; 7 Wageningen University and Research, Lelystad, The Netherlands; 8 Department of Medical Microbiology and Immunology, Medical School, University of Pécs, Pécs, Hungary; 9 University of Texas Medical Branch at Galveston, Galveston, Texas, USA; 10 Friedrich-Loeffler-Institut, Greifswald-Insel Riems, Riems, Germany; 11 University of Oxford, England and University of Edinburgh, Scotland, UK

**Keywords:** hepatitis E virus, *Hepeviridae*, ICTV report, taxonomy

## Abstract

The family *Hepeviridae* includes enterically transmitted small quasi-enveloped or non-enveloped positive-sense single-stranded RNA viruses infecting mammals and birds (subfamily *Orthohepevirinae*) or fish (*Parahepevirinae*). Hepatitis E virus (genus *Paslahepevirus*) is responsible for self-limiting acute hepatitis in humans; the infection may become chronic in immunocompromised individuals and extrahepatic manifestations have been described. Avian hepatitis E virus (genus *Avihepevirus*) causes hepatitis–splenomegaly syndrome in chickens. This is a summary of the International Committee on Taxonomy of Viruses (ICTV) Report on the family *Hepeviridae*, which is available at www.ictv.global/report/hepeviridae.

## Virion

**Fig. 1.**
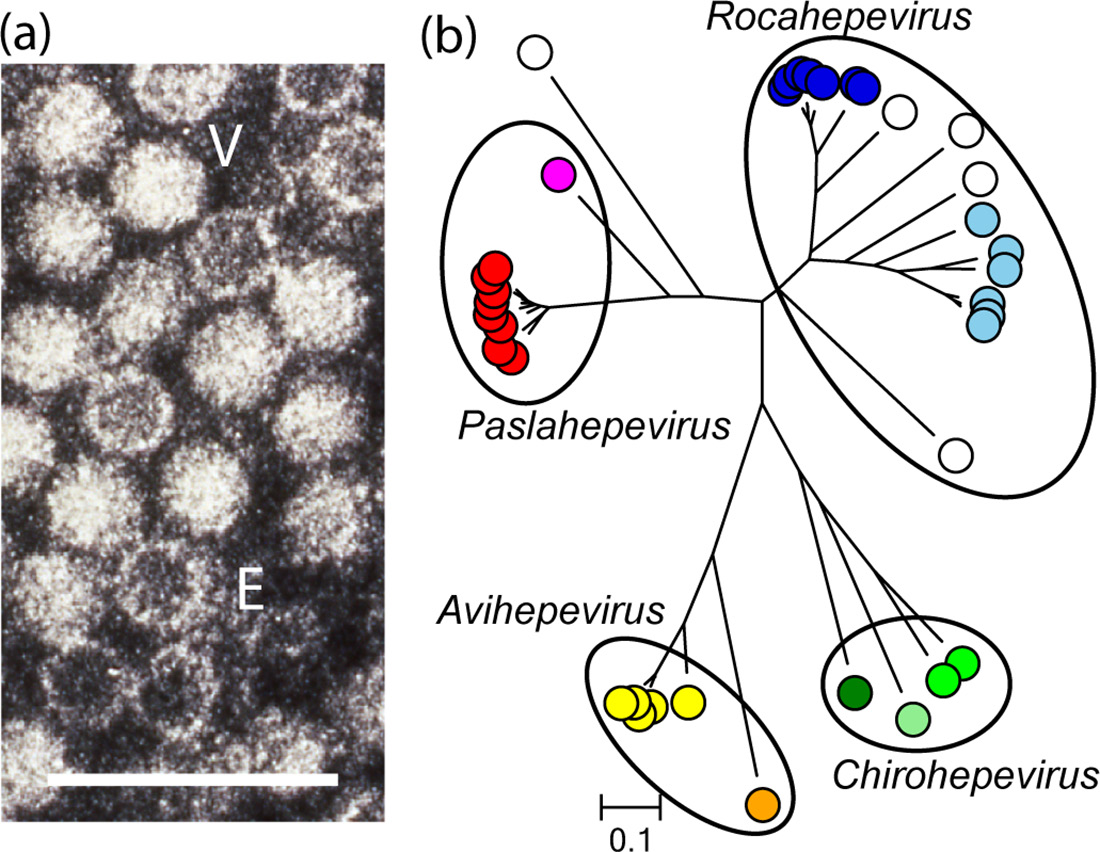
(a) Negative-contrast electron micrograph of human hepatitis E virus particles from a case stool collected in Nepal. (V) virion and (E) empty capsid. Bar: 100 nm (photograph M. Purdy). (b) Phylogeny of orthohepevirin methyltransferase domains (details: ICTV Report).

Virions of human hepatitis E virus are icosahedral, quasi-enveloped (blood or tissue culture [1]) or non-enveloped (faeces), spherical particles with a diameter of 27–34 nm (Table 1, Fig. 1a). The capsid is formed by capsomeres consisting of homodimers of a single capsid protein, forming the virus shell. Each capsid protein contains three linear domains forming distinct structural elements: S (the continuous capsid), P1 (three-fold protrusions) and P2 (two-fold spikes). Neutralizing epitopes have been found in the P2 domain. Each domain contains a putative polysaccharide-binding site that may interact with cellular receptors [2].

**Table 1.** Characteristics of members of the family *Hepeviridae*

Example:	human hepatitis E virus Burma (M73218), species *Paslahepevirus balayani,* genus *Paslahepevirus*
Virion	Quasi-enveloped or non-enveloped, 27–34 nm diameter with a single capsid protein
Genome	6.4–7.2 kb capped positive-sense monopartite RNA containing three ORFs
Replication	Occurs in association with the host endoplasmic reticulum
Translation	From genomic (ORF1) and a subgenomic (ORF2 and ORF3) capped mRNA
Host range	Mammals (*Chirohepevirus, Paslahepevirus, Rocahepevirus*), birds (*Avihepevirus*) and salmonid fishes (*Piscihepevirus*)
Taxonomy	Realm *Riboviria*, kingdom *Orthornavirae*, phylum *Kitrinoviricota*, class *Alsuviricetes*, order *Hepelivirales*: two subfamilies, multiple genera and species

## Genome

**Fig. 2.**
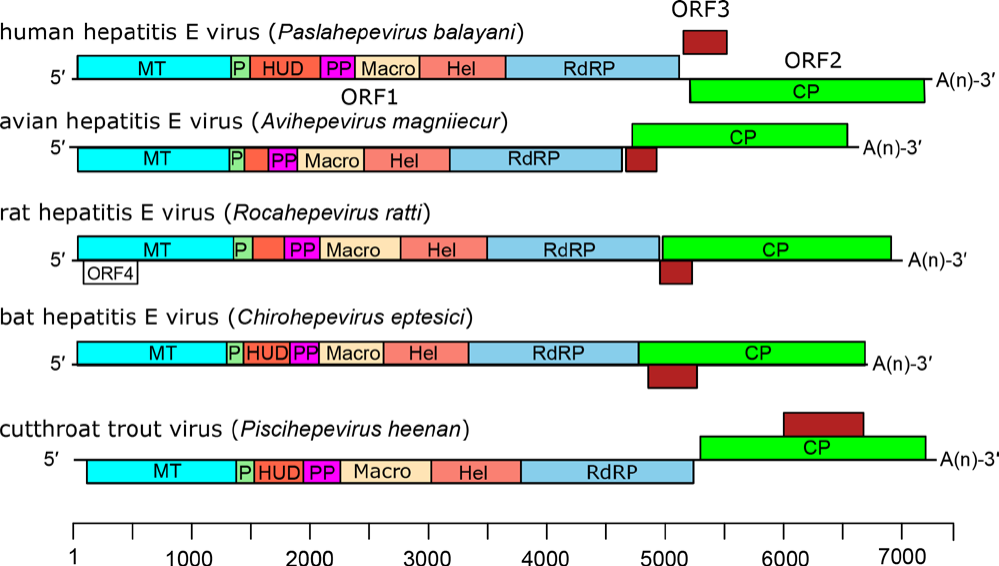
Genome organization of hepeviruses. There are short 5′- and 3′-non-coding regions. The ORF1 polyprotein includes the putative functional domains: MT, methyltransferase; P, a putative papain-like cysteine protease; HUD, hepevirus unique (or Z-) domain; PP, a hypervariable polyproline region; Macro, macro domain; Hel, helicase; and RdRP, RNA-directed RNA polymerase. ORF2 encodes the capsid protein (CP) and ORF3 a small phosphoprotein.

Viral genomes (Fig. 1b) are positive-sense monopartite RNA of 6.4–7.2 kb, with three ORFs flanked by short 5′- and 3′-terminal non-coding regions: ORF2 (capsid protein) overlaps ORF3 but neither overlaps ORF1. The 5′-end is m^7^G-capped and the 3′-end is polyadenylated (Fig. 2). Non-structural proteins encoded by ORF1 have limited similarity with the ‘alpha-like supergroup’ of viruses and contain domains consistent with a methyltransferase, papain-like cysteine protease, macro domain, RNA helicase and RNA-directed RNA polymerase [3]. A small immunoreactive protein (12.5 kDa) encoded by ORF3 has been shown to exhibit multiple functions associated with virion morphogenesis, egress and viral pathogenesis. The capsid and ORF3 proteins are translated from a subgenomic RNA.

## Replication

The viral RNA-directed RNA polymerase associates with the host endoplasmic reticulum through residues encoding a predicted transmembrane domain. Replication involves temporal separation and alternating cycles of synthesis of positive- and negative-sense RNAs [3,4].

## Taxonomy

Current taxonomy: ictv.global/taxonomy. Members of the subfamily *Orthohepevirinae* infect humans and domestic and wild mammals, (genera *Paslahepevirus* and *Rocahepevirus*), bats (*Chirohepevirus*), and birds (*Avihepevirus*) [5,6]. Human hepatitis E virus can cause self-limiting acute hepatitis in humans and is transmitted by contaminated water, the consumption of undercooked or raw meat or iatrogenically through blood transfusion or organ transplantation. Globally, human hepatitis E virus is a major cause of acute hepatitis. Chronic hepatitis E virus infection has increasingly become an important clinical problem in immunocompromised individuals. Cutthroat trout virus, a member of the subfamily *Parahepevirinae*, infects trout and salmon, although its pathogenicity and full host range are unknown.

## Resources

Full ICTV Report on the family *Hepeviridae* : www.ictv.global/report/hepeviridae.
